# The Role of Online Support Groups in the Management of Trichotillomania Symptoms

**DOI:** 10.1002/jclp.70214

**Published:** 2026-08-20

**Authors:** Dan Jenkins, Rizwana Roomaney

**Affiliations:** ^1^ Department of Psychology Stellenbosch University Stellenbosch Western Cape South Africa

**Keywords:** coping, hair‐pulling, qualitative, support groups, trichotillomania

## Abstract

**Background:**

Many individuals with trichotillomania find themselves unable to access effective treatment and are forced to manage symptoms independently. Support groups play an important role in symptom management.

**Aims:**

This qualitative study aimed to explore experiences with online support groups among hair‐pulling individuals (*N* = 20; 10 women, 7 men, 3 non‐binary).

**Methods:**

Data were collected online via semi‐structured interviews. Reflexive thematic analysis yielded four themes: (1) group usage, (2) detrimental group effects, (3) group benefits, and (4) attitudes towards groups.

**Results:**

A major advantage of online groups over in‐person support was the ability for participants to join from home. Participants could join without having to share their appearances, bypassing much of the stigma associated with traditional treatment options. Few participants reported worsening symptoms after joining support groups which they attributed to re‐exposure to pulling triggers, but most had positive views of their groups.

**Conclusions:**

Support groups may play a beneficial role in the management of distress in chronic hair‐pullers.

## 1 Introduction

Trichotillomania is an obsessive‐compulsive and related disorder characterised by repeated hair‐pulling despite efforts to stop (APA 2022). Symptoms typically begin between ages 10 and 13, affecting about 2% of adults in their lifetime (Grant et al. 2021; Grant and Chamberlain 2016). Individuals pull hair from various body sites, most commonly the scalp, eyelashes, eyebrows, facial hair, and pubic areas, often resulting in hair loss (Grant and Chamberlain 2016; Jafferany and Patel 2020). The disorder is associated with psychosocial distress, social and occupational impairment (Anwar and Jafferany 2019; APA 2022; Grant 2019), and feelings of unattractiveness, humiliation, guilt, low self‐worth, and increased depression and anxiety (França et al. 2019; Jafferany and Patel 2020).

### 1.1 Support Groups

Many hair‐pulling individuals (HPIs) turn to support groups to learn about hair‐pulling, manage their symptoms, and find community. Online peer‐to‐peer support groups for mental illnesses such as trichotillomania are increasing (Kaplan et al. 2011). These groups enhance social connectedness, belonging, and personal empowerment (Naslund et al. 2016), and improve mental health through social support and normalising health‐seeking behaviours (Ali et al. 2015; Naslund et al. 2016). They also aid in reducing feelings of isolation and increase connectedness by allowing individuals to compare perspectives (Prescott et al. 2017, 2020), although may induce social comparisons among participants (Tan et al. 2021).

Online support groups are particularly effective for individuals who feel ashamed or unable to discuss their conditions due to stigma (Alvarez‐Jimenez et al. 2016; Bento et al. 2022). For instance, online support groups can challenge stigma, empower individuals, provide hope (Naslund et al. 2016), and offer an alternative to in‐person meetings that may be hindered by shame and social stigma (Balsamo et al. 2023). The anonymity of online groups increases self‐disclosure of health conditions considered to be embarrassing (Rains 2014) and may be beneficial to reducing long‐term distress (Strand et al. 2020). While their impact on clinical symptoms may be limited (Ali et al. 2015; Kaplan et al. 2011), online groups effectively manage the psychosocial consequences of shameful mental health conditions.

Literature on support groups for HPIs is scarce, with most studies focusing on formal therapeutic groups rather than informal peer‐led ones (e.g., Haaland et al. 2017), despite evidence suggesting that informal support groups may reduce symptoms (Toledo et al. 2015). Few studies have examined online peer‐to‐peer support groups for trichotillomania, though members of such groups find them supportive and informative about symptoms, causes, and treatments (Boughn and Holdom 2002; Bruwer and Stein 2005). These studies indicate that online support groups are generally perceived as helpful for social support, normalisation of hair‐pulling experiences, and access to coping strategies, although their impact on symptom reduction is small (Toledo et al. 2015)

A strong social support system significantly impacts the effectiveness of trichotillomania treatment (Everett et al. 2020). Support groups are invaluable for HPIs, offering support, information, and a sense of community. Although cognitive behavioural therapies may reduce symptoms more effectively (Toledo et al. 2015), support groups offer unique benefits by providing a stigma‐free environment (Weingarden and Renshaw 2015). Despite their benefits, research on the use of support groups in managing trichotillomania is limited.

### 1.2 The Present Study

Many people with chronic mental illnesses such as trichotillomania turn to online support groups for community, belonging, and strategies for managing their symptoms and daily challenges (Naslund et al. 2016). These groups provide HPIs a space to openly discuss symptoms they find shameful (Slikboer et al. 2020; Weingarden and Renshaw 2015). Few studies have explored HPIs' experiences with online support groups outside of formal therapy (Boughn and Holdom 2002; Bruwer and Stein 2005; Haaland et al. 2017; Toledo et al. 2015). Unlike prior studies that primarily focussed on descriptive accounts of group use or therapeutic retreats, the present study examines a broader range of experiences, including expectations of groups, their ability to minimise symptoms, and how they are used to manage hair‐pulling outside of formal therapy.

## 2 Methods

### 2.1 Design

This study made use of a cross‐sectional research design and a phenomenological approach. Semi‐structured qualitative interviews were used to explore participants’ experiences with trichotillomania‐focussed online support groups. Interviews were analysed using Reflexive Thematic Analysis (RTA; Braun and Clarke 2019).

### 2.2 Participants and Recruitment

The study recruited participants from the Reddit r/trichotillomania subreddit (https://www.reddit.com/r/trichotillomania) and the TLC Foundation for Body‐Focussed Repetitive Behaviours (BFRBs) website (https://www.bfrb.org). Using purposive sampling, the researchers targeted individuals who (a) self‐reported trichotillomania symptoms, (b) participated in a trichotillomania‐focused online support group, (c) were at least 18 years old, and (d) could engage in an online interview in English. To confirm eligibility, participants were presented with the DSM‐5 criteria for trichotillomania (APA 2022) and asked whether these applied to them. Those who indicated they met these criteria were included in the study, regardless of whether they had received a formal clinical diagnosis. Given the paucity of formal trichotillomania diagnoses due to stigma and treatment avoidance (Grant and Chamberlain 2016; Stewart and Lipner 2020), the study relied on self‐reported symptoms rather than formal diagnoses. Support group advertisements encouraged participants to contact the researchers for study details and informed consent.

To determine an appropriate sample size in lieu of data saturation, we followed Malterud et al. (2016) framework which considers study goals, sample specificity, existing theory, interview quality, and data analysis strategies. Given that the study aim was moderately broad (i.e., seeking to understand HPIs' experiences with online support groups), the sample relatively specific (HPIs who have used online support groups), and the study descriptive rather than generalisable, we judged that a minimum of 15 participants would provide sufficient information power. We opted to use this theory rather than seeking data saturation as in RTA there is often no clear endpoint for data collection beyond the analysts' judgement (Saunders et al. 2018). Ultimately, 23 prospective participants reached out, and 20 returned informed consent forms and were interviewed.

### 2.3 Procedure

A qualitative semi‐structured interview schedule was developed to answer the research question ‘What are HPIs' experiences with online support groups?’. The interview schedule consisted of questions asking how participants discovered the support groups they use, what prompted their use, and whether they feel that the groups have been beneficial to their overall recovery. Dyadic interviews were conducted in a private, online setting by the primary author using Microsoft Teams. Each participant was interviewed separately and in English. All participants indicated availability for follow‐up questions, should any arise. All participants further indicated that they would like to be kept abreast of the outcomes of this study and to be sent publications that arise from their interviews.

Twenty interviews took place between October 2022 and January 2023. Notes were taken during interviews to aid in transcription and analysis. Interviews had a mean length of 47 min. Ethics approval for this study was obtained from Stellenbosch University Health Research Ethics Committee (HREC; ref: S22/08/146). All participants gave consent for their data to be used in this research. Verbal consent was obtained and recorded at the start of each interview. Interviewees received a $10 Amazon gift card as compensation for participation.

### 2.4 Data Analysis

Interviews were conducted using Microsoft Teams video conferencing software (Microsoft Teams 2023) which allowed for video and audio recording and machine‐generated transcription. Transcripts were compared to the recorded audio for accuracy to ensure verbatim English transcription before the original recordings were destroyed. Transcripts were then stripped of personally identifying information (e.g., real names were replaced with pseudonyms of the participants' choice) before being analysed.

Interviews were analysed using an inductive, or bottom‐up, RTA framework (Braun et al. 2019) and using ATLAS.ti 23 software (ATLAS.ti Version 23 2023). While conducting and transcribing interviews, recurrent patterns were noted which later became the basis for themes. Themes were thus derived from the data based on the responses of participants. Coding and theme development were conducted reflexively by the primary author, who brought lived experience with trichotillomania and online support groups to the analysis. Initial codes were generated from quotes in the transcripts and iteratively refined into candidate themes through repeated engagement with the dataset, supported by reflexive notes to monitor interpretive influence. A subset of codes and themes was reviewed by the secondary author as a form of peer debriefing, confirming coherence and comprehensiveness.

### 2.5 Reflexivity

The primary author is a long‐standing member of the Reddit r/trichotillomania and TLC foundation support sites. He became an active member of these groups in seeking community to manage his symptoms. Initially knowing few people with whom he could share his diagnosis, he turned to online support groups and realised that their role in managing trichotillomania is under‐documented in the literature.

Before each interview, the primary author disclosed his membership in the online support groups from which participants were recruited. An informal pre‐interview discussion about the groups helped build rapport, putting participants at ease. Participants appreciated that the researcher had a genuine interest in their success. Although it is possible the researcher had interacted with participants before, the large and anonymous nature of the groups made this unlikely.

Familiarity with the groups' culture and language allowed the primary author to recognise subtle dynamics and meanings that might otherwise have been overlooked. At the same time, his prior experiences with online support groups could have influenced which elements of participants' accounts felt most resonant during coding. To address this, and to strengthen the transparency and trustworthiness of theme development, the author engaged in reflexive note‐taking throughout the analytic process and discussed emerging themes with the secondary author, who brought an external perspective without lived experience of hair‐pulling.

## 3 Findings

### 3.1 Participant Characteristics

Twenty participants provided informed consent and participated in this study. Of these participants, ten self‐identified as women, seven as men, and three as non‐binary. Participants were aged 22–43 years (M = 28.75). Most participants held a bachelor's degree, several were students or worked in healthcare, education, or business‐related fields, and just under half reported being in a relationship. Thirteen participants lived in North America, while the remainder were based in Europe, Africa, or Asia. In terms of support group membership, most were recruited through the Reddit r/trichotillomania community (*n* = 16) or the TLC foundation website (*n* = 4), with a few also participating in smaller national or local trichotillomania groups (*n* = 4). Participant characteristics and support group membership are summarised in Table 1.

**Table 1:** Participant characteristics and support group membership.

**Description**	**No. participants (*n*)**
Gender	
Woman	10
Man	7
Non‐binary	3
Education	
High‐school diploma	5
Bachelor's degree	13
Master's degree	1
Unspecified	1
Occupation	
Student	5
Healthcare and wellness	4
Childcare and education	3
Business/professional services	3
Technology	2
Other/unspecified	3
Relationship status	
Single	6
In a relationship	8
Unspecified	5
Region of residence	
North America	13
Africa	3
Europe	3
Asia	1
Support group membership	
Reddit r/trichotillomania	16
TLC Foundation website	4
Other national/local trichotillomania groups	4
Other online support communities (e.g., autism, LGBTQIA + )	2

### 3.2 Findings from Thematic Analysis

We constructed four themes, each containing several sub‐themes. We named the themes (1) Groups and usage; (2) Detrimental group effects; (3) Group benefits; and (4) Attitudes towards groups.

### 3.3 Theme 1: Groups and Usage

Participants reported seeking online support groups due to a lack of in‐person options. They wanted to share their experiences and seek advice but felt frustrated or ashamed of their symptoms, leaving them with few people to turn to. Sixteen participants mentioned joining support groups because of stigma or not having other to talk to. Some participants began opening up about their pulling behaviour:

[She was] saying [she] has the group that [she] should show me to that I can get information on other things on how people feel. I asked her to introduce me to the group.

– Eric, 25, USA

Participants engaged with support groups in various ways, with some choosing to observe without participating, known as ‘lurking’. Seven participants described themselves as lurkers, with one mentioning that they never participated, while the others hardly ever participated. This anonymous, low‐commitment involvement was appealing, especially for those coming to terms with hair‐pulling or seeking a sense of community. Even casual engagement provided a sense of belonging and reduced feelings of isolation, showing that active participation was seldom necessary to benefit from these groups:

Even though I don't interact I still feel that it does helps because at least in some sense it helps me feel like I'm not the only person who deals with trich.

– Dee, 23, USA

### 3.4 Theme 2: Detrimental Group Effects

Some participants reported negative experiences with online support groups, noting that these groups often failed to provide the immediate relief they sought for hair‐pulling. In total, three participants described disappointment with the lack of a ‘cure’. Other participants observed that new members frequently misunderstood the purpose of these groups, expecting a cure rather than strategies for managing symptoms. When no cure was offered, many became disillusioned and stopped participating. Longstanding members shared management strategies, but they could not offer complete solutions, as the groups were intended for mutual support, not treatment:

When a lot of people are at the very beginning of their journey, … they come to the support group going: ‘How do you stop it?’ And unfortunately, that's not an answer any of us can give.

– Grace, 29, UK

Many participants felt that while online support groups offered social and emotional benefits, they did little to reduce hair‐pulling symptoms. Two participants specifically noted that sharing stories or images could trigger pulling urges, especially for those new to treatment or who pulled under specific conditions. This issue is known among moderators, who may blur photographs or restrict access to certain content unless members request it:

Seeing bald spots or whatever can be a trigger. So, I'm glad that like it'll blur out the image and you don't have to see it unless you want to.

– June, 24, USA

### 3.5 Theme 3: Group Benefits

Despite some negative aspects, participants generally reported overwhelmingly positive experiences with online support groups. Most participants (18 of 20) described the groups as beneficial overall. They found social benefits like support, group healing, and a forum for advice; emotional benefits like reduced isolation, encouragement, and inspiration; and symptom and educational benefits, including better understanding of trichotillomania and their own symptoms, along with a reduction in pulling.

#### 3.5.1 Social and Emotional Benefits

Online support groups provided safe spaces for participants to interact without fear of judgment. Participants could share openly with others who understood their experiences, often for the first time, as they described previously keeping their hair‐pulling secret because of perceived stigma. While most remained anonymous, some shared personal details and photos of their journeys. This connection with others who had similar experiences helped participants feel less alone and supported, improving their psychological and emotional well‐being.

A key emotional benefit of online support groups was providing a sense of normalcy. For example, twelve participants reported that seeing others with trichotillomania helped them feel less shame. Knowing others also experienced hair‐pulling helped participants see their symptoms as external and common, rather than a personal flaw. This realisation reduced feelings of isolation, shame, and self‐stigmatisation, and helped rebuild self‐esteem. Before joining these groups, many participants had no‐one to confide in and explained that they avoided disclosing their hair‐pulling due to others' perceived negative perceptions. The support groups helped alleviate these feelings:

I think that for other people in the group, it was like the first time they were sharing with other people, and it felt really meaningful and significant.

– Remy, 26, USA

#### 3.5.2 Symptom Alleviation and Education Benefits

Participants reported that online support groups helped educate them on managing trichotillomania. Members shared recovery progress, strategies to reduce pulling, and tips for promoting hair growth. Seven participants described learning new coping strategies, and some used the groups to track their progress. The groups provided forums for advice on managing symptoms and emotions. While most groups had formal moderation, strong unofficial moderation was also present, as members discussed beneficial practices and discouraged unhelpful ones. These posts were generally appreciated and seen as inspiring:

She was kind of putting down her story about how she went about that, and that was a motivation for me.

– Josh, 38, UK

Online support groups played a key role in raising awareness of hair‐pulling as a disorder. They provided crucial information for newcomers, with seven participants describing groups that helped them understand their symptoms and recognise pulling as a diagnosable condition rather than a shameful behaviour. For those more familiar with pulling, these groups offered deeper insights into their condition. Participants reported that interactions within the groups helped them accept hair‐pulling as a psychiatric condition rather than a habit. The support groups thus offered valuable educational benefits and support, connecting newer members with more experienced ones:

I've learned different fidgets and stuff that people use [from support groups] … to try and reduce their symptoms. So, I have found it to be beneficial in a lot of ways.

– June, 24, USA

### 3.6 Theme 4: Attitudes Towards Groups

Participants had mixed attitudes toward online support groups. While most (17 of 20) felt these groups positively impacted their management of pulling, their levels of involvement and willingness to recommend them varied. Some preferred to keep their membership private, while others actively sought out these groups for shared experiences and community. Some participants found the groups ineffective or unnecessary, while others appreciated and enjoyed their involvement. Many members tended to ‘lurk’ by viewing content without engaging, which was sufficient for some. The unstructured nature of these groups allowed for both passive and active participation.

Participants sought support and shared experiences in online support groups, which provided spaces to discuss hair‐pulling and its management. However, some found these groups less interactive than hoped, with posts asking for advice often receiving little feedback. This lack of interaction was discouraging, especially for those who preferred to observe rather than actively participate but still relied on community engagement for their journeys.

Some participants were hesitant to join online support groups due to their concerns about privacy or potential negative interactions, with two specifically citing privacy as a barrier. However, those who did join generally found the groups helpful and would recommend them to others. They appreciated how the groups framed hair‐pulling as a diagnosable condition rather than a personal flaw and helped alleviate feelings of isolation and shame. The following participant explains why she would recommend these support groups:

I think that's much easier to do online than in person or where you've got the reduced visibility that you've got the well, the anonymity of it.

– Grace, 29, UK

Ultimately, participants reported positive attitudes towards their online groups and indicated that they were beneficial to their treatment journey, despite feelings of wishing that these groups were not necessary.

## 4 Discussion

We explored twenty HPIs’ experiences of using online support groups in the management of their hair‐pulling symptoms. Participants reported discovering and engaging with their support groups in a variety of ways and noted both positive and negative effects of support groups in their lives. Overall, these groups were described as supportive and a welcome part of the treatment journey and provided participants with much‐needed normalcy and a peer network.

Literature on the use of online support groups for trichotillomania is scarce. The direct effects of online support groups on hair‐pulling are not well‐documented, although existing literature on in‐person support groups (e.g., Toledo et al. 2015) suggests they play a supportive role in the management of trichotillomania. This role appears to be largely social in nature, as even though support groups have a major impact on HPIs' levels of isolation and shame, effects on symptom severity are seldom sustained over time (Slikboer et al. 2020).

In line with literature on the discovery and usage of online support groups (Bruwer and Stein 2005), most participants in this study found support groups by surfing the internet. While referrals to groups are not unheard of, most participants appeared to seek out online support groups individually or discovered them by accident. Most participants interacted with online support groups only occasionally, opting to browse existing posts rather than contribute directly, a trend which has been reported in previous studies of HPI engagement with online support groups (Bruwer and Stein 2005). This does not imply that HPIs hold only a casual interest in support groups, however, as these trends are common across online support groups for other illnesses and diseases (Hu 2017), as well as the internet, regardless of website (Hargittai and Walejko 2008).

Some participants reported negative effects when interacting with online support groups. Slikboer et al. (2020), in a qualitative analysis of HPIs’ experiences with a trichotillomania retreat, noted that some respondents doubted the efficacy of in‐person support groups in managing HP symptoms, particularly in the long term. Similarly, some participants in this study who felt online support groups, though providing social benefits, could not provide the level of relief or change in behaviour they had expected. While online support groups may improve mental health symptoms and psychosocial adjustment (Ali et al. 2015; Naslund et al. 2016; Prescott et al. 2020), full remission from these groups is rare (Ali et al. 2015; Kaplan et al. 2011) and information accuracy can be difficult to gauge (Tan et al. 2021). Most individuals consulting support groups seek interpersonal and social support or disease information (Gumuchian et al. 2019), although some participants in this study expected a cure where traditional treatments had failed. This misalignment of expectations has been long documented in online trichotillomania support groups (Bruwer and Stein 2005) but nevertheless continues to be prevalent in the support groups participants consulted, leaving many feeling disillusioned and helpless.

A small number of participants in this study reported that they pulled hair or relapsed after using support groups. The vast majority of HPIs exposed to support groups or group therapy for trichotillomania report symptom improvements, at least at 6 months post‐treatment, although a small subset of attendees report that symptoms do not improve and that exposure to other HPIs and constant reminders of pulling cause them to relapse (Bruwer and Stein 2005; Slikboer et al. 2020). Similarly, previous studies report increased preoccupation with pulling after online support group use (Tan et al. 2021), possibly due to uncontrolled re‐exposure to triggers (Boughn and Holdom 2002).

Most participants reported several positive experiences with support groups. For example, interacting with other HPIs on support groups allowed participants to gain a sense of community and normalcy in managing hair‐pulling. The social support and the safety and motivation accompanying the use of online support groups echo Slikboer et al.'s (2020) findings, as participants felt that they could freely express difficulties managing hair‐pulling in a safe and non‐judgemental environment. Online groups' capacity to foster caring environments which allow HPIs to share struggles with shame and fear were first documented by Boughn and Holdom (2002), although there remain relatively few support resources available to HPIs (Lochner et al. 2013). A sense of acceptance and belonging, as well as the ability to share experiences with other HPIs, has been identified as being of particular importance to members' assessments of group experiences (Lochner et al. 2013; Slikboer et al. 2020).

Online groups also provided information on hair‐pulling and helped participants understand themselves and their symptoms, in line with existing findings from in‐person trichotillomania retreats (Lochner et al. 2013). Support groups often provided a forum for advice and were a channel through which new coping and management strategies could be learned. Groups often provide more formal psychoeducation about trichotillomania as well, which has been shown to increase knowledge of pulling, reduce HPIs' self‐stigmatisation, loneliness, and shame (Lochner et al. 2013).

Another reported benefit to online support groups was that they combatted feelings of shame and isolation. Their ability to reduce stigma, shame, and feelings of isolation, at least somewhat, is noted in literature (Asplund et al. 2021), and the sense of community and normalcy these groups provide may serve an encouraging and supportive role in coping with hair‐pulling (Haaland et al. 2017). A reduced sense of isolation and the knowledge they are not alone in managing hair‐pulling often leads HPIs in support groups to develop a strong sense of belonging, validation, understanding, and hope (Slikboer et al. 2020), each of which may reduce the stress of hair‐pulling and increase HPIs' quality of life.

To date, few studies have been conducted to explore HPIs’ experiences with online support groups. Many participants reported deriving comfort, normalcy, and reduced isolation simply by reading posts, suggesting that online affordances such as anonymity and flexible participation broaden the reach of support beyond in‐person groups. Unmet expectations, particularly the hope for a cure, remained common, echoing earlier work (Bradley 2021; Bruwer and Stein 2005) and underscoring the importance of framing group purpose and preparing members for realistic outcomes. Participants also noted risks and safeguards unique to digital environments. While sharing stories or images could trigger urges to pull, features such as blurred images or content warnings helped manage exposure, contrary to findings suggesting these create negative anticipatory periods (Bridgland et al. 2019; Simister et al. 2024). Further studies could explore whether these trigger warnings indeed reduce distress in a support group context. Overall, our findings highlight that peer support for trichotillomania reduces isolation and shame while creating a dynamic environment where members negotiate expectations, manage risks, and construct a sense of normalcy.

This study presents with several limitations. Participants reported belonging to a number of both online and in‐person support groups, both for trichotillomania as well as other geographical, health, or sexual identity‐oriented interests. Participants were asked to comment only on their experiences with online support groups for managing hair‐pulling, but it is possible that participants reported general experiences with support groups, and not necessarily online trichotillomania support groups. In addition, as participants reflected on past experiences, their accounts may involve retrospective attribution of effects. As such, it may have been difficult for them to separate which outcomes were specifically linked to their online support group use, introducing a source of bias, particularly if they had longstanding or ongoing involvement.

Recruiting directly from the Reddit and TLC Foundation groups allowed for participants to be reached directly, and so future studies could seek to recruit on a greater number of platforms to develop a more complete picture of how support groups complement the management of trichotillomania. Other studies (e.g., Grant et al. 2024; Pendo et al. 2024) have begun to recruit HPIs directly from support groups, although to date no studies have been conducted to measure whether this introduces a significant source of bias. This potential source of bias may have a profound effect on results as individuals who make frequent use of support groups may be more likely to report positive experiences, whereas those who have would report negative experiences are unlikely to continue using the platforms and thus will be unlikely to respond to recruitment advertisements.

## 5 Conclusion

This study provided a qualitative overview of the experiences of 20 HPIs in using online support groups to manage their hair‐pulling symptoms. This study has expanded upon existing support group knowledge and added to the literature on their usage with trichotillomania. This study forms part of a relatively small group which have sought to explore how patients experienced support groups in the management of obsessive‐compulsive related disorders and provides insights into patient behaviours and how they manage their symptoms outside of traditional healthcare settings.

This study shows that even passive engagement in online groups can foster belonging and reduce stigma, and that unmet expectations around cures may undermine retention. These findings highlight both opportunities and challenges for trichotillomania peer support and suggest directions for research and practice in designing and integrating online groups into care.

## Conflicts of Interest

The authors declare no conflicts of interest.

## Data Availability

The data that support the findings of this study are available on request from the corresponding author. The data are not publicly available due to privacy or ethical restrictions. The data that support the findings of this study are available from the corresponding author, DJ, upon reasonable request. The data are not publicly available due to their containing information that could compromise the privacy of research participants.
